# Effect of Cutting Surface Integrity on Fatigue Properties of TC17 Titanium Alloy

**DOI:** 10.3390/ma16165658

**Published:** 2023-08-17

**Authors:** Dan Wang, Xiyu Chen, Xunqing Lai, Guolong Zhao, Yinfei Yang

**Affiliations:** 1 College of Mechanical and Electrical Engineering, Nanjing University of Aeronautics & Astronautics, Nanjing 210016, China; wonderw@nuaa.edu.cn (D.W.); laiexia@nuaa.edu.cn (X.L.); zhaogl@nuaa.edu.cn (G.Z.); 2 Nanjing High Speed & Accurate Gear (Group) Co., Ltd., Nanjing 211100, China; ycx123146179@163.com

**Keywords:** TC17 titanium alloy, turning, turning parameters, fatigue life

## Abstract

The turning process of titanium alloy material will affect the surface structure of the material and lead to a change in its service life. In this paper, the fatigue behavior of the TC17 titanium alloy turning sample was studied through the bending fatigue test. The fatigue life variation rule under the action of thermal coupling was then discussed. This revealed the fatigue fracture mechanism of TC17; the cracks originated from the surface of the source region, and the transient fault region was a ductile fracture. The mathematical model of turning parameters and surface integrity (roughness, microhardness and residual stress) was established, and the influence of turning parameters on fatigue life was analyzed with a mathematical relationship. Drawing a conclusion, the effects of turning parameters on fatigue life at normal temperature are as follows: Feed > Cutting depth > Cutting speed. The fatigue life of *v_c_* = 30 m/min, *f* = 0.25 mm/r, *a_p_* = 0.3 mm is only 40,586 cycles per week, the fatigue life of *v_c_* = 30 m/min, *f* = 0.05 mm/r, *a_p_* = 0.1 mm has 539,400 cycles per week, that is, the longest fatigue life is 16.6 times the smallest. Small cutting speed, feed, and large cut depth can be chosen based on ensuring practical processing efficiency. The fatigue fracture of the TC17 sample occurred after a certain cycle, and the fatigue fracture mechanism was revealed in this paper.

## 1. Introduction

Titanium alloys are widely used in aerospace fields because of their excellent properties [1,2]. Titanium alloy is a difficult-to-process material as due to low thermal conductivity and high chemical reactivity, the tool wear rate is fast, resulting in higher cutting temperature in the cutting area and poor machinability [3,4]. The turning process causes the crystal lattice of the treated surface material to expand and break, resulting in plastic deformation and a hardened layer, which can change the original properties of the material and affect its service life. So, it is very important to study the influence of the machined surface’s quality on fatigue properties and improve the fatigue life of materials.

According to the existing research conclusions, cutting speed [5], feed [6], and cutting depth [7] have a rough effect on the surface. Because titanium alloy cutting performance is poor [8], it is necessary to study the surface roughness and microhardness of titanium alloy during machining. Mersni and Kalipada et al. [9,10,11,12] analyzed the effects of cutting speed, cutting depth, and feed per tooth on surface roughness, and concluded that feed had the greatest influence on surface roughness. Kumar et al. [13] adopted multi-response grey correlation analysis (GRA) technology to optimize the process parameters, and only observed the effects of cutting parameters on surface roughness and material removal rate through the main-effect diagram. Mazid et al. [14] obtained the optimization parameters of surface roughness ranging from 0.5 um to 1 pm under the conditions of cutting speed ranging from 60 to 250 m/min, feed of 0.1 mm/r and cutting depth of 0.5 mm through turning experiments with different tool radii. Matras et al. [15] proposed that the effect of cutting speed on surface roughness could be ignored and the optimization of process parameters could be carried out with the minimum surface roughness as the goal. Bouuacha et al. [16] used the response-surface method to study the relationship between machining parameters and machining surface roughness, and found that surface roughness is mainly affected by feed and cutting speed. Regarding hardening and microhardness, the hardness of the material after processing is greater on the surface of the material than in the direction of the depth of the material. The study of Ezugwu et al. [17] showed that increasing the feed and cutting depth could improve the microhardness of the surface and subsurface of the material, as well as the depth of the work-hardening zone. Che-Haron and Jawaid [18] studied the surface and subsurface microhardness of Ti-6Al-4V turning and reached similar conclusions. They found that increasing the Cutting speed could obtain a higher hardness value while increasing the feed had little effect.

The above scholars have conducted a lot of studies on the influence of machining parameters on surface roughness, but they are all aimed at a single factor and propose the influence of a single set of parameters on surface roughness.

Concerning the influence of residual stress on fatigue properties, Sharman et al. [19] and Javidi et al. [20] investigated the relationship between surface quality and fatigue properties of TiB2 and 34CrNiMo6 turning, respectively, and held that greater residual stress could improve the fatigue life of materials. Sun et al. [21] studied the effects of different turning parameters on the surface roughness, residual stress, and metamorphic layer of GH4169, and concluded that the surface’s residual compressive stress can improve fatigue performance. Lindemann et al. [22] studied and analyzed the influence of the surface integrity of shot peening and structural changes in the subsurface region on the fatigue properties of γ-TiAl alloy, and concluded that the fatigue properties obtained by shot peening mainly depend on the compressive stress left in the surface region, but are also controlled by the microstructure. According to Syed et al. [23], residual tensile stress will increase the crack propagation rate and thus reduce fatigue life. Kawagoishi et al. [24] believe that the residual compressive stress generated on the surface of the specimen can inhibit the generation and expansion of surface cracks, thus improving the fatigue resistance of the specimen. In summary, a large number of scholars at home and abroad agree that the existence of residual compressive stress can improve the fatigue resistance of parts, while residual tensile stress has the opposite effects.

From the above, it can be inferred that most studies focus on single surface integrity elements such as surface roughness or surface residual stress. Therefore, this paper studies the surface integrity and fatigue properties under the comprehensive influence of several factors. The influence of different machining parameters on surface integrity was analyzed, and the influence mechanism of surface roughness, residual stress and microhardness on fatigue life was studied to explore the mechanism of fatigue failure.

## 2. TC17 Fatigue Test

### 2.1. Sample Preparation and Equipment

The TC17 titanium alloy used in the test was provided by Xi’anYing Yuan Chaojing Electromechanical Equipment Manufacturing Co., Ltd. (Xi’an, China). Its parent material size is 300 × 158 mm, the heat treatment is solution treatment, and the forging billet is freely forged in the same batch. The main components are shown in Table 1.

**Figure 1:**
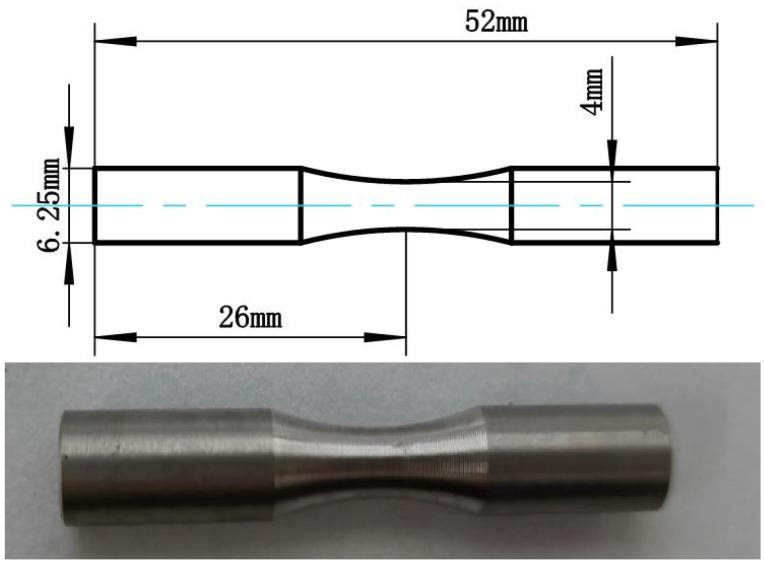
Face turning sample size and physical drawing.

**Figure 2:**
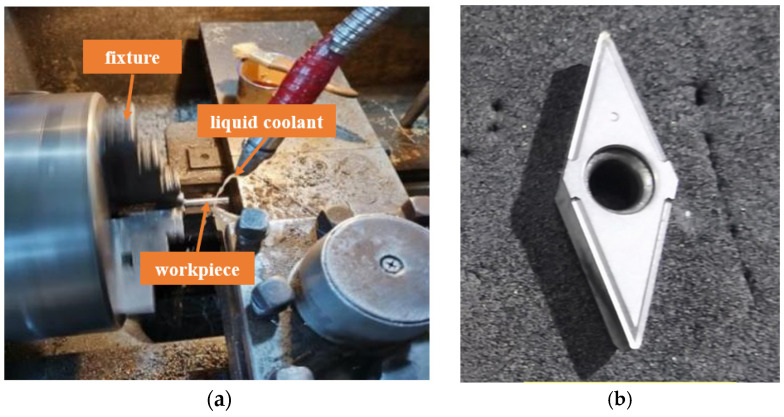
(**a**) Turning machining; (**b**) cutting tool.

To analyze the influence of turning surface integrity on the fatigue life of TC17 titanium alloy, a three-factor, and three-level orthogonal test was designed, and the TC17 fatigue sample was processed by turning technology, and the fatigue life test was carried out at room temperature. The sample size of this test is shown in Figure 1, and the sample material is Ti17 titanium alloy. We chose a carbide blade with a rounded tip radius of 0.4 mm, rake angle of 15° and clearance of 5°. The turning process is carried out on the CA6140 machine tool and the emulsion is cooled. Figure 2a is the turning processing site, and Figure 2b is the turning tool.

### 2.2. Fatigue Test Design

In this experiment, 9 groups of turning fatigue samples with different surface integrity parameters were used for the normal temperature fatigue life test, with 3 pieces in each group. The fatigue life cycle times of different samples were obtained by observing the number of cycles during fracture.

The fatigue test parameters to complete the preparation of the fatigue sample are shown in Table 2.

**Figure 3:**
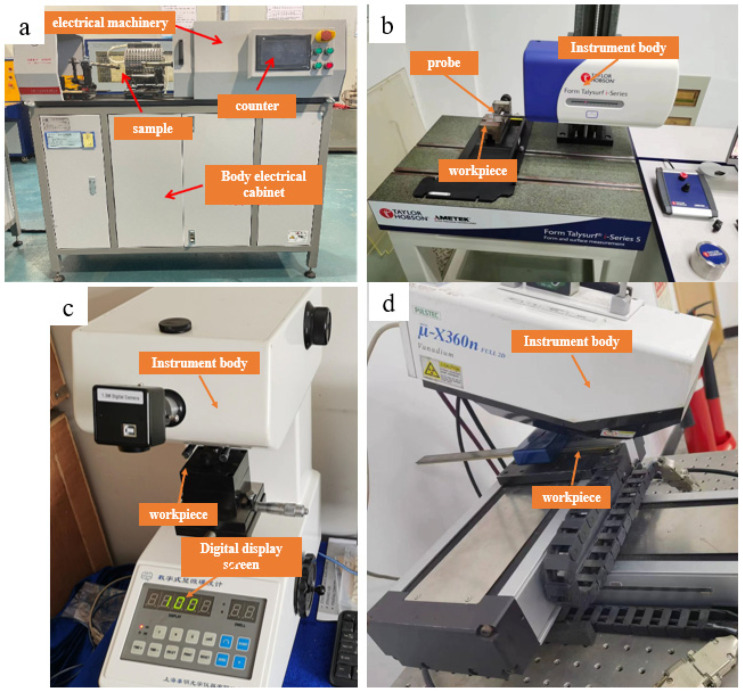
(**a**) QBWP-1000 cantilever bending fatigue testing machine; (**b**) New View 5022 surface profiler; (**c**) automatic microhardness tester; (**d**) μ-X360n X-ray residual stress analyzer.

A QBWP-1000 cantilever bending fatigue testing machine, Changchun Qian Bang Test Equipment Co., Ltd., Changchun, China, was used for the fatigue test; Figure 3a presents the QBWP-1000 cantilever bending fatigue testing machine and the fatigue test conditions are shown in Table 1. The maximum load in the test was selected according to the material manual. The fatigue test under the same turning–processing parameters was repeated three times, and the median fatigue life obtained after data processing was used as the final fatigue test data to reduce the test error of this part. In this paper, New View 5022 surface profiler, ZYGO, CT(Connecticut), America, was used for roughness test, as shown in Figure 3b. The microhardness on the surface of the test rod after turning was measured by an automatic microhardness tester (Qness Q10A+), as shown in Figure 3c. The residual stress on the surface of the test rod after turning was measured by the μ-X360n X-ray residual stress analyzer made by PULSTEC, Hamamatsu, Japan, as shown in Figure 3d.

For ease of understanding, Table 3 lists the notes on the abbreviations used in this article

Generally speaking, the fatigue life dispersion is significant. According to the reliability analysis, the fatigue life distribution law of the test piece under a given stress level can be described by the Weibull distribution and lognormal distribution function; so, a relatively simple lognormal distribution is adopted in this paper to fit the fatigue life data [25].

(1) $$\bar{Y}=\frac{1}{n}\sum_{i=1}^{n}\mathrm{lg}N_{i}$$

where, $\bar{Y}$ is the average logarithmic fatigue life of the sample under the same parameter, *N_i_* is the fatigue life cycle number of the sample, and *n* is the number of fatigue samples in each group of tests, that is, *n* = 3. The median fatigue life *N*_50_ for each group, that is, the fatigue life reliability, is calculated as 50%.

(2) $$\bar{Y}=\mathrm{lg}N_{50}$$

(3) $$N_{50}=10^{\bar{Y}}$$

According to the above formula, the median fatigue life of the material can be calculated, and the influence of surface integrity on the fatigue performance of TC17 titanium alloy can be analyzed. Table 4 shows the surface integrity parameters of these 9 groups.

A TM3000 scanning electron microscope, HITACHI, Tokyo, Japan was used to analyze the fracture morphology of the fractured fatigue sample.

## 3. Results and Discussion

### 3.1. Influence of Turning Parameters on Surface Integrity

#### 3.1.1. Surface Roughness

**Figure 4:**
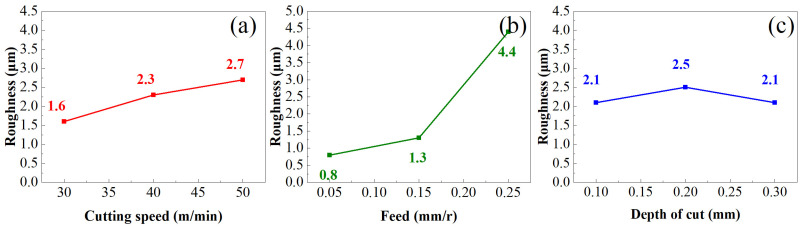
(**a**) Effect of cutting speed on roughness (**b**) Effect of feed on roughness (**c**) Effect of depth of cut on roughness.

Surface roughness is an index to evaluate surface integrity, and its influence on the performance of parts cannot be ignored. Figure 4 shows the influence of turning parameters on surface roughness (*R_a_*), where Ra increases with the increase in cutting speed (*v_c_*), which is because the increase in *v_c_* leads to the strengthening of tool vibration in the cutting process, thus affecting the surface roughness. With the increase in the feed amount *f*, *R_a_* also increases because the larger the *f*, the larger the residual height of the machined surface, and the higher the *R_a_* value. With the increase in cutting depth (*a_p_*), the roughness value (*R_a_*) first increases and then decreases, indicating that the increase in *a_p_* in a certain range has a positive effect on the stability of the machining system. The surface roughness in the feed direction was selected as the research object for analysis and the New View 5022 surface profiler of ZYGO Company was used for testing. The surface profilometer has a maximum scanning depth of 2–150 μm, a lateral resolution of 110 nm, and a vertical resolution of 0.1 nm. The test was measured with a 10× objective lens. Refer to the surface topography measurement method proposed by Han Haitjema [26] and CL Giusca [27] to eliminate the influence of noise and uncertainty. And Przemysław Podulka [28] proposed the frequency- and direction-based method to reduce the influence of surface topography measurement errors.

For orthogonal tests, an exponential mathematical model can be established. If *K_st_* is used to represent the stress concentration coefficient of the machining surface, the empirical model of the influence of turning parameters on the surface stress concentration coefficient is as follows.

(4) $$K_{st}=A_{0}v_{c}^{a}f^{b}a_{p}^{c}$$

In the formula, *A*_0_ is a constant, and *a*, *b*, and *c* are the constant quantities corresponding to the cutting parameters. The fitting was carried out by Matlab, and according to the fitting results by the least-squares method, the linear regression correlation coefficient was obtained, and the fitting relevant parameter values were substituted into the formula as follows:

(5) $$K_{st}=2.0906v_{c}^{0.1383}f^{0.3117}a_{p}^{-0.0709}$$

#### 3.1.2. Microhardness

The surface microhardness can effectively reflect the performance changes of the material’s turning surface. In the turning process, the cutting force and cutting heat caused by different turning parameters will lead to different degrees of plastic deformation in the processing area, resulting in different degrees of work hardening of the material. Therefore, the analysis of the influence of turning parameters on microhardness has a guiding significance for the optimization of tuning parameters.

**Figure 5:**
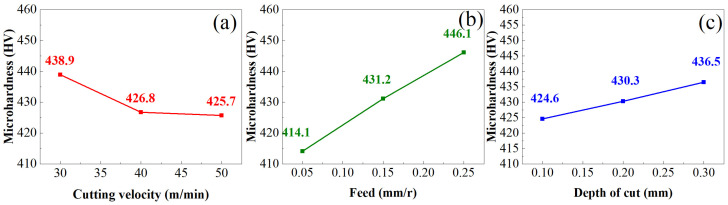
(**a**) Effect of cutting speed on microhardness (**b**) Effect of feed on microhardness (**c**) Effect of depth of cut on microhardness.

Figure 5 shows the influence of turning parameters on surface microhardness (HV), where HV decreases with the increase in *v_c_*, because the increase in *v_c_* will reduce the contact time between the cutting edge and the machined surface, thus weakening the extrusion effect of the turning tool on the machined surface and reducing the occurrence of plastic deformation, resulting in a decrease in the surface hardness of the material. With the increase in *f*, HV gradually increases, which is mainly related to the cutting force generated in the turning process. With the increase in *f*, the cutting force continues to increase, which intensifies the scratching between the turning tool and the machining surface, resulting in the intensification of the plastic deformation of the material. Although the cutting temperature will also rise in this process, the cutting fluid is used to cool the material in this test. Therefore, plastic deformation plays a major role in the strengthening of hardness; so, in general, the increase in *f* will increase the hardness of the material. With the increase in *a_p_*, HV also increases because the increase in *a_p_* represents the material’s removal rate becoming larger, then the cutting force acting on the machining surface is larger, and the plastic deformation becomes larger, thereby increasing the microhardness.

If HV is used to represent surface microhardness, the mathematical equation of the influence of turning parameters on surface microhardness obtained by the linear regression analysis method is as follows:

(6) $$HV=638.4415v_{c}^{-0.0595}f^{0.0307}a_{p}^{0.0661}$$

#### 3.1.3. Residual Stress

Generally speaking, the residual stress on the surface of a part has a certain effect on its fatigue life. The residual compressive stress can inhibit the initiation of cracks and thus increases its fatigue life, while the residual tensile stress has the opposite effect. Because the cutting force and cutting heat are different for parts processed with different turning parameters, the residual stress on the surface of the parts will also be different after machining; so, the analysis of the influence of turning parameters on the residual stress is of guiding significance for the selection of turning parameters in actual production.

**Figure 6:**
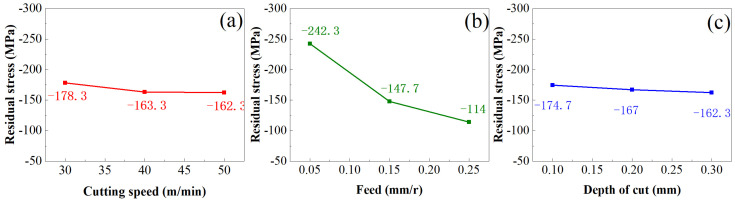
(**a**) Effect of cutting speed on residual stress (**b**) Effect of feed on residual stress (**c**) Effect of depth of cut on residual stress.

Figure 6 shows the influence of turning parameters on surface residual stress (*σ*), where *σ* decreases with the increase in *v_c_*, decreases with the increase in *f*, and decreases with the increase in *a_p_*. The main reasons for the above trend change are the following: the increase in *v_c_*, the contact time between the cutting edge of the turning tool and the machining surface becomes shorter, the effect of the turning tool on the workpiece weakened, and the degree of plastic deformation is reduced, which leads to the decline in *σ*. The increase in *f* will increase by cutting force, resulting in the change in the cutting tool’s removing-material angle, and the cutting force will produce tensile stress, thereby offsetting part of the compressive stress and reducing *σ*. The increase in *a_p_* will cause the cutting temperature to rise, and the residual stress will be generated after the workpiece is heated and expanded, which will reduce the *σ*.

If *σ* is used to represent the surface’s residual compressive stress, the mathematical equation of the influence of turning parameters on the surface residual compressive stress obtained by the linear regression analysis method is as follows:

(7) $$\left|\sigma \right|=250.073v_{c}^{-0.2595}f^{-0.5071}a_{p}^{0.1283}$$

### 3.2. Fatigue Life Analysis

Fatigue fracture is the main failure form of metal materials in service. The factors affecting the fatigue life of the workpiece include not only the property of the workpiece material itself, the size and direction of the applied stress, but also the surface integrity of the workpiece, and different processing processes that have an impact on the surface integrity. This paper mainly studies the influence of different turning parameters on the fatigue life of TC17.

**Figure 7:**
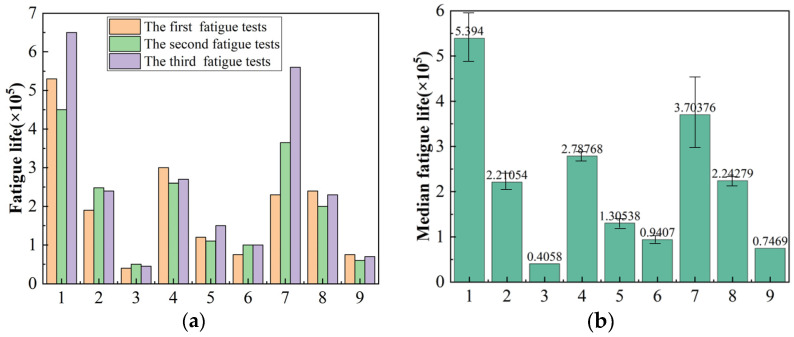
(**a**) Fatigue life under different machining methods. (**b**) The median fatigue life under different machining methods.

Figure 7 shows the fatigue life of TC17 titanium alloy fatigue samples prepared under different turning parameters under normal temperature conditions. It can be seen from the figure that the fatigue life of the TC17 titanium alloy at normal temperature is more than 104 weeks, and the numerical dispersion is relatively large. The fatigue life of *f* = 0.25 mm/r, *a_p_* = 0.3 mm is only 40,586 cycles per week, and the fatigue life of *v_c_* = 30 m/min, *f* = 0.05 mm/r, *a_p_* = 0.1 mm has 539,400 cycles per week under the first group of turning parameters, that is, the longest fatigue life is 16.6 times that of the smallest.

**Figure 8:**
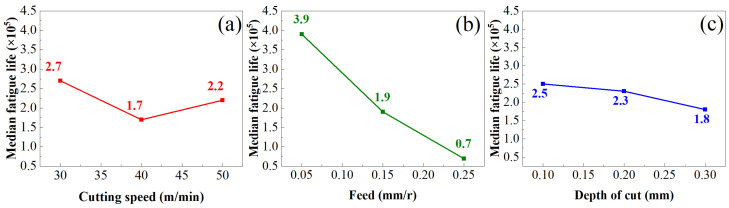
(**a**) Effect of cutting speed on fatigue life (**b**) Effect of feed on fatigue life (**c**) Effect of depth of cut on fatigue life.

Figure 8 shows the influence of three turning parameters (*v_c_*, *f*, and *a_p_*) on the fatigue life of TC17. The following can be seen: With the increase in *v_c_*, the fatigue life first decreases and then increases. This is because, with the increase in *v_c_*, the cutting heat generated in the cutting process increases the cutting temperature, resulting in a thermal softening effect on the cutting surface, reducing the degree of work hardening on the surface, accelerating the initiation rate of cracks on the metal surface and accelerating the crack propagation, resulting in a decline in the fatigue life of the sample. With the further increase in *v_c_*, the cutting force will continue to increase, and the cutting force will offset the thermal softening effect caused by the cutting heat, so that the degree of hardening of the machined surface will increase, thus inhibiting the surface cracks of the material and improving the fatigue life of the sample. *f* has a great influence on the fatigue life of TC17. Within a given range, the fatigue life becomes smaller with an increase in *f*. This is because, with the increase in *f*, the residual machining-tool marks on the turning surface are uneven, the contour height of the machined surface is large, and the increase in surface roughness results in a serious stress concentration caused by surface defects. The fatigue life of the test part is reduced. The fatigue life decreases with the increase in *a_p_* because with the increase in *a_p_*, the overall roughness of the machined surface shows an increasing trend; the rougher the machined surface topography, the more defects on the machined surface, the crack source on the surface increases correspondingly, and the fatigue life of the test piece decreases accordingly.

The stress concentration coefficient *K_st_*, microhardness HV and surface residual stress σ can be calculated according to Formulas (5)–(7). The defects in the manufacturing process and the random characteristics such as machine vibration in the turning process affect the surface quality of the sample. At the same time, during the formation of the machined surface, the residual stress will also occur when the hardening phenomenon occurs, that is, the fatigue life change in the TC17 titanium alloy turning test parts should be comprehensively considered with the coupling effect between various factors. Therefore, a mathematical model between fatigue life *N_50_* and stress concentration coefficient *K_st_*, microhardness HV and surface residual stress σ can be established using linear regression analysis.

(8) $$N_{f}=A_{0}K_{t}^{a}{\mathrm{HV}}^{b}\sigma_{r}^{c}$$

where N_f_ is fatigue life and A_0_ is constant. The fitting was carried out by Matlab, and the correlation coefficient of linear regression was 0.90125 according to the fitting result by the least-squares method. According to the fitting value of the relevant parameters, it was substituted into the formula as follows.

(9) $$N_{f}=10^{-5.7873}K_{\mathrm{st}}^{-1.0253}{\mathrm{HV}}^{2.6031}{\left|\sigma_{r}\right|}^{1.03541}$$

According to the above formula, it can be seen that microhardness has the greatest influence on the fatigue life of the TC17 titanium alloy turning sample, and the stress concentration coefficient and residual stress have little influence. The fatigue life will be improved by increasing the microhardness of the turning surface. The residual compressive stress can delay the fatigue crack generation and early crack propagation on the surface of the specimen; so, the increase in the residual compressive stress on the surface of the metal material will also increase fatigue life. When there are small defects on the surface of the sample, the surface stress concentration is serious, which accelerates the initiation of fatigue cracks and reduces the fatigue life of the sample.

According to the previous section, the mathematical formulas of surface microhardness, residual compressive stress, stress concentration coefficient, and turning parameters have been obtained, and the empirical model of fatigue life at room temperature can be obtained by combining the two.

(10) $$N_{f}=7908.3821v_{c}^{-0.1356}f^{-1.0225}a_{p}{}^{0.5228}$$

From the above formula, it can be seen that the sensitivity degree of influence on the fatigue life of TC17 titanium alloy at room temperature is as follows: Feed > Cutting depth > Cutting speed, and the law obtained is consistent with the surface-quality parameters, so the fatigue life of TC17 titanium alloy under different turning parameters can be predicted according to the above formula.

This method can estimate the fatigue life of the TC17 titanium alloy test rod well, but there are still some shortcomings, such as the fact that the actual use environment and the test cycle environment will be different, and the noise and humidity in the environment will have a certain impact.

### 3.3. Analysis of Fatigue Fracture

#### 3.3.1. Macroscopic Morphology of Fatigue Fracture

**Figure 9:**
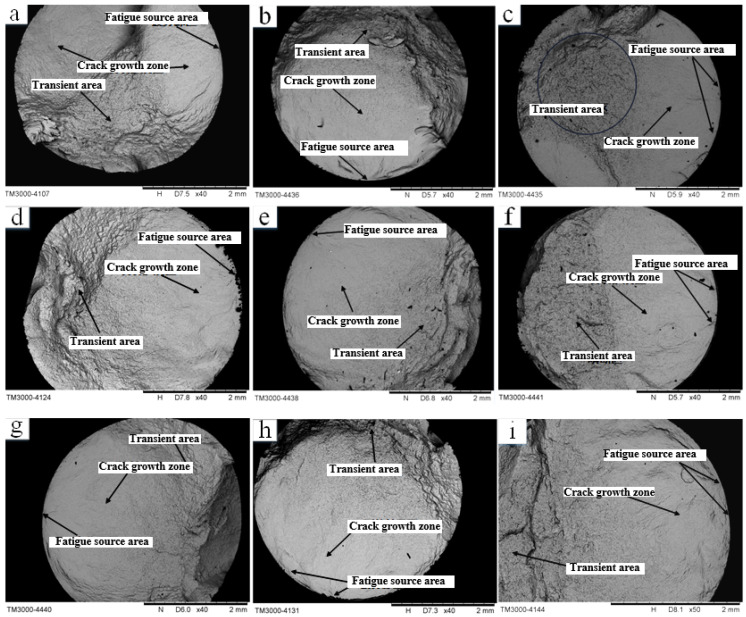
(**a**) The first group of parameters fatigue fracture morphology (**b**) The second group of parameters fatigue fracture morphology (**c**) The third group of parameters fatigue fracture morphology (**d**) The fourth group of parameters fatigue fracture morphology (**e**) The fifth group of parameters fatigue fracture morphology (**f**) The sixth group of parameters fatigue fracture morphology (**g**) The seventh group of parameters fatigue fracture morphology (**h**) The eighth group of parameters fatigue fracture morphology (**i**) The ninth group of parameters fatigue fracture morphology (**h**) The first group of parameters fatigue fracture morphology.

Generally, the fatigue crack propagation of materials can be divided into three parts: the fatigue source region, the expansion region and the transient fracture region. Figure 9 shows the macroscopic morphology of the fatigue fracture of samples processed with different turning parameters at normal temperature. The non-uniformity of the surface material after turning is the main factor leading to fatigue cracks. In the fatigue test, the fatigue crack first starts from the source region, and then radially expands to the center. When the rest of the crack is unable to bear the applied load, the material will shear and break instantaneously. It can be seen from the figure that the fatigue fracture of the TC17 titanium alloy sample is relatively smooth near the fatigue-source region, and the end face away from the source region is not smooth, and the roughness is increasing. There are multiple fatigue source regions in fatigue fracture at normal temperature. The main reason for this phenomenon is the existence of contour pits on the surface of turning, and the stress concentration at the pits will promote the initiation of fatigue cracks. Through the macroscopic fracture analysis, it can be seen that the cracks are generated from the surface of the sample, and no internal cracks are found. No inclusions were found in the initiation zone of the fracture, and no “fisheye” was found, which indicates that the main cause of fatigue fracture failure of the sample is its material problem.

#### 3.3.2. Microstructure of Fatigue Fracture

Fatigue fracture is generally divided into brittle fracture and plastic fracture (ductile fracture); the former is the fracture behavior of the sample without obvious plastic deformation, the latter is the sample before the fracture incurs a large number of plastic deformation. In the microscopic range, the ductile fracture has an obvious deformation zone, forming tearing or appearing as a cavity-aggregation fracture, and cleavage will occur on the cleavage plane of brittle fracture.

**Figure 10:**
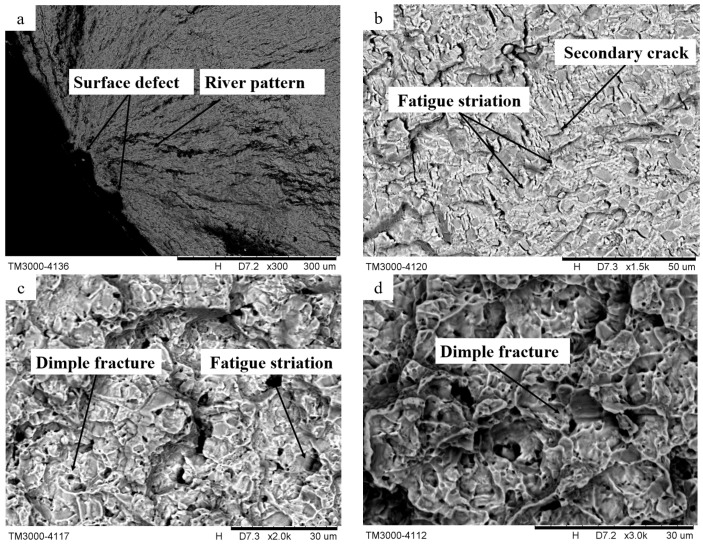
(**a**) Crack source region; (**b**) spread region; (**c**) junction between spread region and transient fault region; (**d**) transient fault region.

Figure 8 shows the microscopic morphology of fatigue fracture at normal temperature. From the micro-topography of the crack source region in Figure 10a, it can be seen that there are obvious wear marks. When the fatigue life of the sample is long, the wear marks are generated by repeated contact and running of the matching surface of the crack in the source region under the action of repeated rotation and bending deformation. The crack sources in the figure all start from the surface, indicating that the surface integrity of the fatigue sample significantly affects its fatigue life. In the figure, the crack initiation occurs at the defect of the turning surface, and the surface defect causes the stress concentration effect there, which promotes the generation and propagation of the surface crack. At the same time, the river pattern can be seen near the source area, that is, the fatigue source area shows the characteristics of cleavage fracture, which belongs to the characteristics of brittle fracture. Figure 10b shows the microstructure of the crack growth area at normal temperature, where secondary cracks and fatigue bands can be observed. The fatigue bands are composed of a series of approximately parallel stripes perpendicular to the crack propagation direction, and each fatigue stripe represents a fatigue-loading cycle. The number of fatigue bands reflects the cycle times of fatigue cyclic load, and the spacing of fatigue bands can reflect the level of fracture stress. Therefore, the width of fatigue bands and the number of fatigue bands indicates that the fatigue-crack growth rate is slow. The direction of the secondary crack in the figure is basically parallel to the fatigue strip, which is a secondary crack exposed on the fracture of the sample. Its formation may be due to the fact that the TC17 titanium alloy used in this test has a mesh structure, and the anisotropy of the crystal and different sliding systems cause the uneven deformation of the alloy. When the crack extends to the junction of the α phase and β phase of the alloy, a large stress concentration effect is generated, which eventually leads to its formation. Figure 10c shows the microscopic morphology of the junction between the extended zone and the transient zone of the fracture. It can be observed that there are fatigue bands and interlinked pits, namely dimples. A dimple is a typical ductile fracture characteristic, which is generated at the defects of the matrix material under the action of external cyclic load stress, and grows under the complex stress at the crack tip. When it breaks, it generates a void and gathers with the surrounding micro-cracks to form a hole, and the hole will continue to grow, increase in value and cause fracture. Figure 10d is the transient fracture zone of the fracture, and it can be observed that there are a certain number of dimples in the figure, and the fracture surface of the dimples in the transient fracture zone has the microscopic characteristics of a ductile fracture.

## 4. Conclusions

In this paper, the fatigue test of part of TC17 was processed by turning technology, and the rotating fatigue test was carried out at room temperature to investigate the influence of turning parameters on the surface integrity and fatigue life of the sample, and the fatigue-fracture morphology was studied at room temperature. The main conclusions are as follows:

(1) The rule of influence of turning parameters on surface integrity was studied, the mathematical equation between the two was summarized, the mathematical formula of surface integrity on fatigue life of materials at normal temperature was fitted according to the linear regression equation, the rule of influence on fatigue life at normal temperature within the given range of turning parameters was studied, and the mathematical equation of fatigue life of turning parameters was fitted.
(2) The biggest influence on fatigue life is the microhardness of the machined surface, followed by the stress concentration coefficient and residual stress. Fatigue life increases with the increase in microhardness and residual compressive stress, and decreases with the increase in stress concentration coefficient. At normal temperature, fatigue life decreases with the increase in feed, increases with the increase in cutting depth, decreases first and then increases with the increase in cutting speed, and the sensitivity of turning parameters, in descending order, is feed, cutting depth and cutting speed.
(3) The fatigue fracture morphology of the TC17 titanium alloy was analyzed, and its fracture mechanism was obtained. At normal temperature, cracks start from the surface of the sample, and no internal crack initiation is found. Secondary cracks and fatigue bands can be observed in the crack-expansion zone. With the increase in residual compressive stress, the initiation of the fatigue crack source can be reduced, which is conducive to the improvement of fatigue life, and the selection of turning parameters under actual machining is considered comprehensively.

## Figures and Tables

**Table 1:** Chemical composition of TC17 titanium alloy.

Ti	Al	Sn	Zr	Mo	Cr	N	Fe	C	O	Others
Bal.	4.9	2.0	1.9	4.2	4.2	0.02	0.10	0.02	0.10	Each < 0.10 Total < 0.30

**Table 2:** Fatigue test parameters.

Test Parameters	*σ*_max_	*r*	*f*	*Tn*	Waveform	Failure Judgment
Set value	720 MPa	−1	83.3 Hz	20 °C	Sine wave	Working section fracture

**Table 3:** This paper uses a list of nouns and acronyms.

Full Name	Surface Roughness	Microhardness	Residual Stress	Cutting Speed	Feed	Cut Depth
Abbreviation	*R_a_*	HV	*σ*	*v_c_*	*f*	*a_p_*

**Table 4:** Surface integrity parameters of fatigue samples.

*No.*	*v*_c_ (m/min)	*f* (mm/r)	*a_p_* (mm)	*R*_a_ (μm)	*Std.* (μm)	*σ* (MPa)	*Std.* (MPa)	*HV*	*Std.*
01	30	0.05	0.1	0.34	0.064	−249	10.756	405.1	20.543
02	30	0.15	0.2	0.95	0.052	−162	8.523	440.2	31.953
03	30	0.25	0.3	3.51	0.563	−124	8.498	471.5	27.146
04	40	0.05	0.2	0.77	0.022	−237	9.275	421.9	30.523
05	40	0.15	0.3	1.12	0.298	−155	2.543	437.3	35.148
06	40	0.25	0.1	4.99	1.205	−98	1.988	421.1	25.857
07	50	0.05	0.3	1.45	0.377	−241	6.726	429.5	27.288
08	50	0.15	0.1	1.93	0.326	−126	12.287	416.1	19.244
09	50	0.25	0.2	4.68	1.022	−120	16.283	431.5	5.248

## Data Availability

Not applicable.
